# Incidence and risk factors of pneumococcal pneumonia in adults: a population-based study

**DOI:** 10.1186/s12890-023-02497-2

**Published:** 2023-06-08

**Authors:** Olga Ochoa-Gondar, Verónica Torras-Vives, Cinta de Diego-Cabanes, Eva M. Satué-Gracia, Angel Vila-Rovira, María J. Forcadell-Perisa, Domingo Ribas-Seguí, Clara Rodríguez-Casado, Angel Vila-Córcoles

**Affiliations:** 1 Primary Health Care Service “Camp de Tarragona”, Institut Català de la Salut, Tarragona, Spain; 2 Unitat de Suport a la Recerca of Tarragona, Institut Universitari d’Investigació en Atenció Primària Jordi Gol (IDIAP Jordi Gol), Tarragona, Spain; 3 Information System for the Improvement of Research in Primary Care (SIDIAP), Primary Care Research Institute Jordi Gol, Universitat Autonoma de Barcelona, Barcelona, Spain

**Keywords:** Incidence, Pneumococcal pneumonia, Risk factors, Multimorbidity, Adults

## Abstract

**Background:**

Infection caused by Streptococcus pneumoniae, mainly invasive pneumococcal disease (IPD) and pneumococcal pneumonia (PP), are a major public health problem worldwide. This study investigated population-based incidence and risk of PP among Catalonian persons ≥ 50 years-old with and without specific underlying conditions/comorbidities, examining the influence of single and multi-comorbidities in the risk of suffering PP.

**Methods:**

Population-based cohort study involving 2,059,645 persons ≥ 50 years-old in Catalonia, Spain, who were retrospectively followed between 01/01/2017-31/12/2018. The Catalonian information system for development of research in primary care (SIDIAP) was used to establish baseline characteristics of the cohort (comorbidities/underlying conditions), and PP cases were collected from discharge codes (ICD-10: J13) of the 68 referral Catalonian hospitals.

**Results:**

Global incidence rate (IR) was 90.7 PP cases per 100,000 person-years, with a 7.6% (272/3592) case-fatality rate (CFR). Maximum IRs emerged among persons with history of previous IPD or all-cause pneumonia, followed by haematological neoplasia (475.0), HIV-infection (423.7), renal disease (384.9), chronic respiratory disease (314.7), liver disease (232.5), heart disease (221.4), alcoholism (204.8), solid cancer (186.2) and diabetes (159.6). IRs were 42.1, 89.9, 201.1, 350.9, 594.3 and 761.2 in persons with 0, 1, 2, 3, 4 and ≥ 5 comorbidities, respectively. In multivariable analyses, HIV-infection (hazard ratio [HR]: 5.16; 95% CI: 3.57–7.46), prior all-cause pneumonia (HR: 3.96; 95% CI: 3.45–4.55), haematological neoplasia (HR: 2.71; 95% CI: 2.06–3.57), chronic respiratory disease (HR: 2.66; 95% CI: 2.47–2.86) and prior IPD (HR: 2.56; 95% CI: 2.03–3.24) were major predictors for PP.

**Conclusion:**

Apart of increasing age and immunocompromising conditions (classically recognised as high-risk conditions), history of prior IPD/pneumonia, presence of chronic pulmonary/respiratory disease and/or co-existing multi-comorbidity (i.e., two or more underlying conditions) are major risk factors for PP in adults, with an excess risk near to immunocompromised subjects. Redefining risk categories for PP, including all the above-mentioned conditions into the high-risk category, could be necessary to improve prevention strategies in middle-aged and older adults.

## Background

Infections caused *by Streptococcus Pneumoniae*, mainly invasive pneumococcal disease (IPD) and pneumococcal pneumonia (PP), are a major cause of morbidity and mortality around the world. Young children, individuals with at-risk or immunocompromising conditions and elderly people support the greatest burden of pneumococcal disease, with higher incidence and mortality in these persons [1].

The incidence of IPD (which includes mainly bacteremic PP, but also pneumococcal meningitis, sepsis and non-focal bacteremias) is well documented, with an incidence of approximately 10–50 IPD cases per 100,000 person-years in developed countries. [1–4] However, the true incidence of PP (which includes bacteremic PP but mostly non-bacteremic PP) is not well known considering difficulties in characterizing non-bacteremic PP cases. [3, 5] Indeed, there is very large difference between PP incidence rates (ranging from 68 to 7000 cases per 100,000 person-years) reported by different studies [6].

Regarding risk factors to suffer pneumococcal infections, it is well documented that some conditions such as anatomical or functional asplenia, immunocompromised status, presence of chronic illnesses (e.g., chronic pulmonary/respiratory disease, heart disease, diabetes mellitus), high-risk behaviours (e.g., alcoholism and/or smoking) and low socioeconomic status are well recognised risk factors for suffering IPD. [1, 2, 7] However, data on risk factors for suffering PP is scarce in the literature. On this concern, the existing population-based epidemiological data on PP must to be updated considering that the role of pneumococcus as causative pathogen of pneumonia could have decreased in recent years (after routine anti-pneumococcal vaccination use). [8, 9] Of note, apart of the recognised role of the above mentioned underlying risk conditions, few is known about the possible effect of multiple concurrent risk conditions (multi-comorbidity) on the risk of suffering IPD/PP [10].

The present study aims to update population-based incidence and risk of hospitalisation from PP during 2017–2018 in the Catalonian general population ≥ 50 years-old examining the influence of different specific underlying medical conditions/comorbidities on the risk of suffering PP. We also assessed the role of concurrent multi-comorbidity on the risk of PP. Data assessing pneumococcal vaccination effectiveness in the same study cohort have been previously published. [11, 12].

## Methods

### Design, setting and study population

This is a population-based retrospective cohort study involving 2,059,645 Catalonian middle-aged and older adults, who were all persons ≥ 50 years-old (birth day date before 01/01/1967) affiliated to the 274 Primary Care Centres (PCCs) managed by the Catalonian Health Institute (ICS, *Institut Catala de la Salut*) around Catalonia (Spain).

In Catalonia (a Spanish region with 7.5 million people) there are 358 PCCs, of which 274 (76.5%) are managed by the ICS and 84 are managed by other providers. The analysed cohort (n = 2,059,645 persons ≥ 50 years) represented a 72.6% of the total 2,838,002 Catalonian inhabitants in this age strata according to census data on January 2017 [13]. In the study setting, pneumococal vaccines are routinely administered to the elderly (the 23-valent pneumococcal polysaccharide vaccine [PPsV23] publicly funded since the 2000s), high-risk individuals (PPsV23 and/or 13-valent pneumococcal conjugate vaccine [PCV13], publicly funded since 2012) and infants (universal free PCV13 publicly funded since 2016) [14].

Cohort members were followed since the study start date (01/01/2017) until the occurrence of any event, death, disenrollment from the PCC, or until the end of two-year follow-up (31/12/2018). The study was approved by the ethical committee of the Institution (Ethics Committee IDIAP Jordi Gol, file 20/065-PCV) and was conducted in accordance with the general principles for observational studies [15].

### Data sources

The “Information System for the Development of Research of the Primary Care” (SIDIAP) of Catalonia, [16, 17] which compiles administrative data and clinical information contained in the electronic PCC’s medical records (coded by the International Classification of Diseases, 10th Revision, ICD-10) was used to identify demographic characteristics, comorbidities and underlying medical conditions of cohort members at baseline.

To identify study events (hospitalisations from PP) occurred among cohort members across the study period, we used the national surveillance system for hospital discharge data (“*Conjunto Mínimo Básico de Datos*”, CMBD). The CMBD System, maintained by the Spanish Ministry of Health, includes 98% of Spanish hospitals, encompassing an estimated 99.5% of the Spanish population (covered in the National Health Care System by a compulsory health insurance) [18]. In the present study we used CMBD hospital discharge codes, coded according to the ICD-10 codes, reported during 2017 and 2018 from the 68 Catalonian hospitals. Methodology linking both SIDIAP and CMBD databases before analyses has been extensively described elsewhere [11].

### Outcome definitions

Pneumococcal pneumonia (PP) was defined on the basis of hospital discharge codes reported by the CMBD in hospitalisations occurred among cohort members from January 1, 2017 to December 31, 2018 (ICD-10 code J13, any listed position). According institutional guidelines, [19] participating hospitals applied similar diagnoses checklist and treatment for patients with a clinical suspicion of pneumonia (which is established on the basis of an acute respiratory illness, with evidence of a new infiltrate in a chest radiograph), being blood/sputum cultures and urinary antigen testing used as conventional diagnostic techniques (performed according to the attending physician in each case). Code J13 is assigned on the basis of a laboratory-confirmed diagnosis (i.e., positive blood/sputum culture or urinary antigen test). Case-fatality was considered when the patient deceased (by any cause) within hospital stay. Deaths from any cause occurred among cohort members across study period were captured by administrative data (vital status), which is periodically updated in the SIDIAP database.

### Covariables

Baseline covariables were age, sex, history of hospitalisation from IPD or all-cause pneumonia during previous two-years, pneumococcal vaccination (PCV13/PPsV23) and influenza vaccination status, presence of high-risk/immunocompromising conditions (asplenia, immunodeficiency, HIV infection, chronic severe renal disease, solid organ or haematological neoplasia and/or immunosuppressive treatment), and presence of at-risk conditions (chronic pulmonary/respiratory disease, chronic heart disease, diabetes mellitus, chronic liver disease, alcoholism and smoking). Criteria used to define underlying risk conditions are described in the Appendix.

### Statistical analyses

Incidence rates (IRs) were calculated as person-years, considering that the numerator was the number of events and the denominator was the sum of the persons-time contributed to each cohort member during the study period. Only a first episode of hospitalisation from pneumonia during the study period was considered and, therefore, IRs do not include multiple events per person. Confidence intervals (CIs) for the IRs were calculated assuming a Poisson distribution for uncommon events. Chi-squared or Fisher’s tests, as appropriate, were used to calculate p-values in the comparison of categorical variables.

Cox regression analyses were used to calculate unadjusted and multivariable-adjusted hazards ratios (HRs) and evaluate the association between baseline conditions and the time to the first outcome during the study period [20]. We developed two regression analysis: in the first (model 1) specific comorbidities/risk conditions were considered individually; in the second (model 2) we considered multi-comorbidity (number of comorbidities). The final multivariable models were adjusted by all significant variables plus pneumococcal and influenza vaccine status (which were judged epidemiologically relevant variables). All results were expressed with 95% CIs. Statistical significance was set at p < 0.05 (two-tailed). Data was analysed by using IBM SPSS Statistics for Windows, version 24 (IBM Corp., Armonk, N.Y., USA).

## Results

### Characteristics of the study cohort

The study cohort included 2,059,645 individuals, 951,011 (46.2%) men and 1,108,634 (53.8%) women, with a mean age of 66 years-old (Standard Deviation: 11.4) at baseline. By age groups, 1,040,009 (50.5%) cohort members were 50–64 years-old, 689,342 (33.5%) were 65–79 years-old and 330,294 (16%) were 80 years or older.

Overall, 1,055,206 (51.2%) cohort members were healthy subjects (persons without comorbidities/underlying risk conditions), 800,992 (38.9%) were at-risk subjects (immunocompetent persons with at least one at-risk condition) and 203,447 (9.9%) were high-risk subjects (one or more immunocompromising condition).

Considering multi-comorbidity, 668,608 (32.5%) cohort members had one comorbidity/risk condition alone, 244,543 (11.9%) had two, 70,688 (3.4%) had three, 16,851 (0.8%) had four and 3749 (0.2%) had five or more comorbidities/risk conditions. Table 1 shows baseline characteristics of cohort members (prevalence of specific comorbidities/underlying risk conditions and vaccinations’ history) according to age strata.

**Table 1:** Baseline characteristics of the study cohort

Age	50–64 yrs	65–79 yrs	≥ 80 yrs	Overall
Characteristics	(N = 1,040,009) n (%)	(N = 689,342) n (%)	(N = 330,294) n (%)	(N = 2,059,645) n (%)
**Sex:**				
Men Women	512,122 (49.2) 527,887 (50.8)	317,234 (46) 372,108 (54)	121,655 (36.8) 206,639 (63.2)	951,011 (46.2) 1,108,634 (53.8)
**Hospitalization history**:				
IPD in prior 2 yrs All-cause pneumonia in prior 2 yrs	203 3250 (0.3)	299 6068 (0.9)	230 (0.1) 7449 (2.3)	732 (0.1) 16,767 (0.8)
**Vaccination history**: PPsV23 (at any time) PCV13 (in previous 5 yrs) Flu vaccine (in prior autumn)	95,585 (9.2) 4400 (0.4) 120,794 (11.6)	434,832 (63.1) 6128 (0.9) 319,620 (45.4)	268,131 (81.2) 3389 (1.0) 212,207 (64.2)	798,548 (38.8) 13,917 (0.7) 652,621 (31.7)
Asplenia	226 (< 0.1)	108 (< 0.1)	28 (< 0.1)	362 (< 0.1)
HIV infection	3372 (0.3)	458 (0.1)	56 (< 0.1)	3886 (0.2)
Primary immunodeficiency	406 (< 0.1)	277 (< 0.1)	115 (< 0.1)	798 (0.1)
Severe renal disease	2534 (0.2)	6496 (0.9)	13,506 (4.1)	22,536 (1.1)
Haematological neoplasia	2051 (0.2)	3092 (0.4)	1834 (0.6)	6977 (0.3)
Solid neoplasia	32,380 (3.1)	49,296 (7.2)	29,699 (9.0)	111,375 (5.4)
Immunosuppressive therapy	24,215 (2.3)	33,440 (4.9)	24,650 (7.5)	82,305 (4)
Chronic respiratory disease	64,032 (6.2)	84,348 (12.2)	52,316 (15.8)	200,696 (9.7)
Chronic heart disease	41,316 (4.0)	93,424 (13.6)	84,942 (25.7)	219,682 (10.7)
Diabetes mellitus	101,728 (9.8)	161,308 (23.4)	88,251 (26.7)	351,287 (17.1)
Alcoholism	39,945 (3.8)	21,387 (3.1)	3686 (1.1)	65,018 (3.2)
Smoking	264,177 (25.4)	69,430 (10.1)	10,933 (3.3)	344,540 (16.7)
**No. of comorbidities**:				
0 1 2 3 4 ≥5	595,274 (57.2) 325,606 (31.3) 90,664 (8.7) 22,188 (2.1) 5123 (0.5) 1154 (0.1)	331,669 (48.1) 225,057 (32.6) 94,348 (13.7) 29,11 (4.2) 7335 (1.1) 1822 (0.3)	128,263 (38.8) 117,945 (35.7) 59,531 (18.0) 19,389 (5.9) 4393 (1.3) 773 (0.2)	1,055,206 (51.2) 668,608 (32.5) 244,543 (11.9) 70,688 (3.4) 16,851 (0.8) 3749 (0.2)

NOTE: Comparing differences by age, all differences were statistically significant with p < 0.001 (chi-squared test) except for primary immunodeficiency (p = 0.426)

### Time follow-up, number of events, incidence and case-fatality rates

Across study period, 83,440 (4.1%) cohort members died and 51,175 (2.5%) moved or were lost subjects. Overall, cohort members were observed for a total of 3,958,528 person-years.

An amount of 3592 cohort members had a first episode of hospitalisation from PP across study period (1997 [55.6%] men and 1595 [44.4%] women; 761 [21.2%] in 50–64 years old, 1313 [36.6%] in 65–79 years old and 1518 [42.3%] in 80 years or older). Considering baseline-risk strata, 865 PP cases (24.1%) occurred in healthy subjects, 1850 (51.5%) in at-risk persons and 877 (24.4%) in immunocompromised/high-risk persons.

Specifically, 1169 PP cases (32.5%) had a history of chronic pulmonary/respiratory disease, 1051 (29.3%) diabetes mellitus, 885 (24.6%) chronic heart disease, 604 (16.8%) were smokers, 435 (12.1%) had cancer (377 [10.5%] solid cancer and 58 [1.6%] haematological neoplasia), 427 (11.9%) had received immunosuppressive therapy in the previous 12 months, 251 (7.0%) had alcoholism, 177 (4.9%) liver disease, 139 (3.9%) severe renal disease, 31 (0.9%) HIV infection, and 5 (0.1%) other immunodeficiencies (Table 2).

**Table 2:** Characteristics of pneumococcal pneumonia (PP) cases as compared with healthy subjects

Outcome	With PP	Without PP	p value
	(N = 3592)	(N = 2056053)	
Characteristics	**N (%)**	**N (%)**	
**Age:**			
50–64 yrs	761 (21.2)	1,039,248 (50.5)	< 0.001
65–79 yrs	1313 (36.6)	688,029 (33.5)	
≥ 80 yrs	1518 (42.3)	328,776 (16.0)	
**Sex**:			
Men	1997 (55.6)	949,014 (46.2)	< 0.001
Women	1595 (44.4)	1,107,039 (53.8)	
**Hospitalization history**:			
IPD in prior 2 yrs	26 (0.7)	706 (< 0.1)	< 0.001
All-cause pneumonia in prior 2 yrs	316 (8.8)	16,451 (0.8)	< 0.001
**Vaccination history**:			
PPsV23 (at any time)^*^	2426 (67.5)	796,122 (38.7)	< 0.001
PCV13 (in previous 5 yrs)^**^	87 (2.4)	13,830 (0.7)	< 0.001
Flu vaccine (in prior autumn)	1928 (53.7)	650,693 (31.6)	< 0.001
Asplenia	2 (0.1)	360 (< 0.1)	0.085
HIV infection	31 (0.9)	3855 (0.2)	< 0.001
Primary immunodeficiency	5 (0.1)	793 (< 0.1)	0.002
Severe renal disease	139 (3.9)	22,397 (1.1)	< 0.001
Haematological neoplasia	58 (1.6)	6919 (0.3)	< 0.001
Solid neoplasia	377 (10.5)	110,998 (5.4)	< 0.001
Immunosuppressive therapy	427 (11.9)	81,878 (4.0)	< 0.001
Chronic respiratory disease	1169 (32.5)	199,527 (9.7)	< 0.001
Chronic heart disease	885 (24.6)	218,797 (10.6)	< 0.001
Chronic liver disease	177 (4.9)	40,746 (2.0)	< 0.001
Diabetes mellitus	1051 (29.3)	350,236 (17.0)	< 0.001
Alcoholism	251 (7.0)	64,767 (3.2)	< 0.001
Smoking	604(16.8)	343,936 (16.7)	0.889
**No. of comorbidities**			
0	865 (24.1)	1,054,341 (51.3)	< 0.001
1	1156 (32.2)	667,452 (32.5)	
2	915 (25.5)	243,628 (11.8)	
3	441 (12.3)	70.247 (3.4)	
4	169 (4.7)	16,682 (0.8)	
≥5	46 (1.3)	3703 (0.2)	

NOTE: IPD (invasive pneumococcal disease); PPsV23 (23-valent pneumococcal polysaccharide vaccine); PCV13 (13-valent pneumococcal conjugate vaccine)

^*^Fifty pneumococcal pneumonia cases had received PPsV23 after study start

^**^Forty-four pneumococcal pneumonia cases had received PCV13 after study start

With regard to multi-comorbidity, 865 (24.1%) Of the total 3592 PP cases had no comorbidities, 1156 (32.2%) had one comorbidity alone, 915 (25.5%) had two coexisting comorbidities, 441 (12.3%) had three, 169 (4.7%) had four and 46 (1.3%) had five or more comorbidities.

Global IR was 90.7 PP cases per 100,000 person-years (95% CI: 85.2–96.5). By sex, IRs were 109.7 in men and 74.6 in women. IRs substantially increased by age (37.3 in 50–64 years, 98.3 in 65–79 years and 259.8 in ≥ 80 years) and baseline-risk strata (42.0, 120.7 and 238.6 in healthy, at-risk and high-risk subjects, respectively).

The greatest incidence emerged among those cohort members with history of hospitalisation from IPD or all-cause pneumonia within previous two-years (2258.9 and 1223.4 per 100,000 person-years, respectively). Considering specific comorbidities, maximum IRs (per 100,000 person-years) appeared among persons with haematological neoplasia (475.0), followed by persons with HIV infection (423.7), severe renal disease (384.9), primary immunodeficiency (332.7), chronic pulmonary/respiratory disease (314.7), immunosuppressive treatment (286.6), liver disease (232.5), chronic heart disease (221.4), alcoholism (204.8), solid cancer (186.2), diabetes mellitus (159.6) and smoking (90.4) (Table 3).

**Table 3:** Time follow-up, absolute number of pneumococcal pneumonia (PP) events and incidence rates (IRs) by distinct underlying conditions

Parameter	No. subjects at baseline	Time follow-up* (person/yrs)	Hospitalisation for
			pneumococcal pneumonia
			**Events IR** ^*^ **95% CI** ^**^
**Age**:			
50–64 yrs	1,040,009	2,038,432	761 37.3 (34.8–40.0)
65–79 yrs	689,342	1,335,884	1313 98.3 (92.3-104.6)
≥ 80 yrs	330,294	584,212	1518 259.8 (244.0-276.4)
**Sex**:			
Men	951,011	1,819,859	1997 109.7 (103.0-116.7)
Women	1,108,634	2,138,669	1595 74.6 (70.0-79.4)
**Hospitalization history**:			
IPD in prior 2 yrs	732	1151	26 2258.9 (1475-3320.6)
All-cause pneumonia in prior 2 yrs	16,767	25,830	316 1223.4 (846.6-1371.4)
**Vaccination history**:			
PPsV23 (at any time)^***^	798,548	1,532,186	2476 161.6 (138.0-189.2)
PCV13 (in previous 5 yrs) ^****^	13,917	33,228	131 394.2 (332.7- 466.8)
Flu vaccine (in prior autumn)	652,621	1,233,701	1928 156.3 (133.5–183.0)
HIV infection	3886	7316	31 423.7 (286.0-605.9)
Primary immunodeficiency	798	1503	5 332.7 (133.4-685.4)
Severe renal disease	22,536	36,116	139 384.9 (324.8-455.7)
Haematological neoplasia	6977	12,201	58 475 (366.0-618.0)
Solid neoplasia	111,375	202,440	377 186.2 (167.4-207.1)
Immunosuppressive therapy	82,305	148,967	427 286.6 (261.1-314.7)
Chronic respiratory disease	200,696	371,417	1169 314.7 (295.5-334.8)
Chronic heart disease	219,682	399,807	885 221.4 (207.2-236.5)
Chronic liver disease	40,923	76,121	177 232.5 200.4-269.7)
Diabetes mellitus	351,287	658,711	1051 159.6 (149.9-169.8)
Alcoholism	65,018	122,586	251 204.8 (180.6-232.2)
Smoking	344,540	667,933	604 90.4 (83.3–98.0)
**No. of comorbidities**:			
0	1,055,206 (51.2)	2,057,817	865 42.0 (39.3–44.9)
1	668,608 (32.5)	1,285,621	1156 89.9 (72.7-112.3)
2	244,543 (11.9)	454,973	915 201.1 (174.6-215.6)
3	70,688 (3.4)	125,638	441 351.0 (315.5-390.3)
4	16,851 (0.8)	28,437	169 594.3 (507.5-695.9)
≥5	3749 (0.2)	6043	46 761.2 (554.9–1020.0)

NOTE: IPD (invasive pneumococcal disease); PPsV23 (23-valent pneumococcal polysaccharide vaccine); PCV13 (13-valent pneumococcal conjugate vaccine); IR (incidence rate); CI (confidence interval)

^*^IR denotes incidence rates per 100,000 person/years

^**^Confidence intervals were calculated assuming a Poisson distribution for uncommon events

^***^Besides 786,946 PPsV23 vaccinated persons at baseline, 46,325 persons received PPsV23 after study start (contributing to the analyses with 33,176 person-years as unvaccinated and 13,080 person-years as PPsV23 vaccinated)

^****^Besides 5010 PCV13 vaccinated persons at baseline, 4460 persons received PCV13 after study start (contributing to the analyses with 2260 person-years as PCV13 unvaccinated and 2153 person-years as PCV13 vaccinated)

IRs dramatically increased with the increasing number of baseline comorbidities, being 42.1, 89.9, 201.1, 350.9, 594.3 and 761.2 in persons with 0, 1, 2, 3, 4 and ≥ 5 comorbidities, respectively.

Overall case-fatality rate (CFR) was 7.6% (272/3592). By age groups, CFRs were 4.5% (34/761) in 50–64 years, 5.9% (77/1313) in 65–79 years and 10.6% (161/1518) in ≥ 80 years (p < 0.001). CFR did not significantly differ by gender, being 8% (160/1997) in men vs. 7% (112/1595) in women (p = 0.265). CFRs were greater among patients with haematological neoplasia (10.3%), severe renal disease (10.1%), chronic heart disease (9.3%) or solid neoplasia (8.5%) and were relatively lower among patients with diabetes mellitus (7.6%), alcoholism (6.4%), liver disease (6.2%), smoking (5.8%) and chronic pulmonary/respiratory disease (5.7%) (Table 4). CFR did not significantly vary by number of comorbidities, being 9.6%, 6.4%, 6.8%, 8.2%, 7.1% and 10.9% among patients with 0, 1, 2, 3, 4 and ≥ 5 comorbidities, respectively.

**Table 4:** Case-fatality rates (CFRs) by distinct underlying conditions

Parameter	No. of cases	No. of deaths	Case-fatality rate	
			%	(95% CI)
**Age**:				
50–64 yrs	761	34	4.5	3.2–6.2
65–79 yrs	1313	77	5.9	4.7–57.3
≥ 80 yrs	1518	161	10.6	9.2–12.3
**Sex**: Men	1997	160	8	6.9–9.3
Women	1595	112	7	5.9–8.4
**History of IPD in prior 2 yrs**	26	2	7.7	2.1–24.4
**All-cause pneumonia in prior 2 yrs**	316	22	7	4.6–10.3
**PCV13 vaccinated**	131	8	6.1	3.1–11.6
**PPsV23 vaccinated**	2476	211	8.5	7.5–9.7
**Flu vaccinated**	1928	176	9.1	7.9–9.1
**HIV infection**	31	0	0	0–11.0
**Primary immunodeficiency**	5	1	20	3.6–62.5
**Severe renal disease**	139	14	10.1	6.1–16.2
**Haematological neoplasia**	58	6	10.3	4.8–20.8
**Solid neoplasia**	377	32	8.5	6.1–11.7
**Immunosuppressive treatment**	427	33	7.7	5.6–10.7
**Chronic respiratory disease**	1169	66	5.7	4.5–7.1
**Chronic heart disease**	885	82	9.3	7.5–11.4
**Chronic liver disease**	177	11	6.2	3.5–10.8
**Diabetes mellitus**	1051	80	7.6	6.2–9.4
**Alcoholism**	251	16	6.4	4.0-10.1
**Smoking**	604	35	5.8	4.2-8.0
**No. of comorbidities**:				
0	865	83	9.6	7.8–11.7
1	1156	74	6.4	5.1-8.0
2	915	62	6.8	5.3–8.6
3	441	36	8.2	6.0-11.1
4	169	12	7.1	4.1–12.0
≥5	46	5	10.9	4.7–23.0

NOTE: IPD (invasive pneumococcal disease); PPsV23 (23-valent pneumococcal polysaccharide vaccine); PCV13 (13-valent pneumococcal conjugate vaccine); CFR (case-fatality rate); CI (confidence interval)

### Risk for pneumococcal pneumonia

Table 5 shows Cox regression (model 1) evaluating the influence of different specific underlying conditions on the risk of hospitalisation from PP in the studied cohort. In the multivariable-adjusted analysis, HIV infection (HR: 5.16; 95% CI: 3.57–7.46), prior all-cause pneumonia (HR: 3.96; 95% CI: 3.45–4.55), haematological neoplasia (HR: 2.71; 95% CI: 2.06–3.57), chronic pulmonary/respiratory disease (HR: 2.66; 95% CI: 2.47–2.86) and history of prior IPD (HR: 2.56; 95% CI: 2.03–3.24) were the underlying conditions most strongly associated with an increasing risk of PP. In addition, age/years (HR: 1.06), sex male (HR: 1.33), alcoholism (HR: 1.84), severe liver disease (HR: 1.79), immunosuppressive treatment (HR: 1.76), smoking (HR: 1.58), chronic renal disease (HR: 1.42), chronic heart disease (HR: 1.31), diabetes mellitus (HR: 1.25) and solid cancer (HR: 1.18) were also associated with a statistically significant increased risk. To have received flu vaccine in prior autumn (HR: 1.02), PCV13 within the previous five years (HR: 1.24) and/or PPsV23 at any time (hr: 1.07) did not appear associated with a reduced risk.

**Table 5:** Cox regression analysis evaluating risk of PP by distinct specific underlying conditions (model 1)

	Unadjusted		Multivariable-adjusted	
	HR (95% CI)	p value	HR (95% CI)	p value
**Age (continuous, yrs)**	1.07 (1.06–1.08)	< 0.001	1.06 (1.05–1.07)	< 0.001
**Sex male**	1.47 (1.38–1.57)	< 0.001	1.33 (1.25–1.43)	< 0.001
**History of IPD in prior 2 yrs**	23.70 (19.39–28.97)	< 0.001	2.56 (2.03–3.24)	< 0.001
**All-cause pneumonia in prior 2 yrs**	14.48 (12.90-16.25)	< 0.001	3.96 (3.45–4.55)	< 0.001
**HIV infection**	4.70 (3.30–6.69)	< 0.001	5.16 (3.57–7.46)	< 0.001
**Primary immunodeficiency**	3.67 (1.53–8.82)	0.004	1.93 (0.80–4.65)	0.143
**Severe renal disease**	4.32 (3.64–5.11)	< 0.001	1.42 (1.19–1.69)	< 0.001
**Haematological cancer**	5.28 (4.07–6.84)	< 0.001	2.71 (2.06–3.57)	< 0.001
**Solid cancer**	2.17 (1.95–2.41)	< 0.001	1.18 (1.05–1.32)	0.006
**Immunosuppressive therapy**	3.43 (3.10–3.79)	< 0.001	1.76 (1.59–1.96)	< 0.001
**Chronic pulmonary disease**	4.65 (4.33–4.98)	< 0.001	2.66 (2.47–2.86)	< 0.001
**Chronic heart disease**	2.90 (2.69–3.13)	< 0.001	1.31 (1.21–1.42)	< 0.001
**Chronic liver disease**	2.64 (2.27–3.07)	< 0.001	1.79 (1.53–2.10)	< 0.001
**Diabetes Mellitus**	2.07 (1.92–2.22)	< 0.001	1.25 (1.16–1.35)	< 0.001
**Alcoholism**	2.35 (2.07–2.67)	< 0.001	1.84 (1.61-0.2.11)	< 0.001
**Smoking**	1.00 (0.91–1.09)	0.939	1.58 (1.43–1.74)	< 0.001
**PPsV23 (at any time)**	3.40 (3.16–3.64)	< 0.001	1.07 (0.98–1.18)	0.153
**PCV13 (in previous 5 yrs)**	3.63 (2.99–4.40)	< 0.001	1.24 (1.00-1.52)	0.046
**Flu vaccine (in prior autumn)**	2.55 (2.39–2.73)	< 0.001	1.02 (0.94–1.10)	0.675

NOTE: IPD (invasive pneumococcal disease); PPsV23 (23-valent pneumococcal polysaccharide vaccine); PCV13 (13-valent pneumococcal conjugate vaccine); HR (hazard ratio); CI (confidence interval)

Table 6 shows a supplementary Cox regression analysis (model 2) assessing the role of multimorbidity (number of baseline comorbidities) on the risk of suffering PP. As compared with persons without comorbidities, multimorbidity increased substantially the risk of HPP in our study population, with multivariable-adjusted HRs increasing from 1.76 (95% CI: 1.61–1.92) in persons with one comorbidity alone up to 3.10 (95% CI: 2.81–3.41) for two comorbidities, 4.66 (95% CI: 4.14–5.26) for three comorbidities, 7.07 (95% CI: 5.96–8.38) for four comorbidities and 8.70 (95% CI: 6.43–11.76) for five or more comorbidities.

**Table 6:** Cox regression analysis evaluating risk of PP by number of multicomorbidities (model 2)

	Unadjusted		Multivariable-adjusted	
	HR (95% CI)	p value	HR (95% CI)	p value
**Age**:				
50–64 yrs	1.00 (reference)	< 0.001	1.00 (reference)	< 0.001
65–79 yrs	2.66 (2.44–2.90)		1.95 (1.77–2.16)	
≥ 80 yrs	6.51 (5.97–7.11)		4.46 (4.01–4.96)	
**Sex men**	1.47 (1.38–1.57)	< 0.001	1.35 (1.27–1.45)	< 0.001
**Prior IPD**	23.70 (19.39–28.97)	< 0.001	2.64 (2.09–3.34)	< 0.001
**Prior all-cause pneumonia**	14.48 (12.90-16.25)	< 0.001	4.36 (3.80–5.01)	< 0.001
**PPsV23 (at any time)**	3.40 (3.16–3.64)	< 0.001	1.09 (0.99–1.20)	0.075
**PCV13 (in previous 5 yrs)**	3.63 (2.99–4.40)	< 0.001	1.48 (1.21–1.80)	< 0.001
**Flu vaccine in prior autumn**	2.55 (2.39–2.73)	< 0.001	1.01 (0.93–1.09)	0.878
**No. of comorbidities**				
0	1.00 (reference)	< 0.001	1.00 (reference)	< 0.001
1	2.14 (1.96–2.33)		1.76 (1.61–1.92)	
2	4.77 (4.35–5.24)		3.10 (2.81–3.41)	
3	8.30 (7.40–9.31)		4.66 (4.14–5.26)	
4	14.02 (11.89–16.53)		7.07 (5.96–8.38)	
≥5	17.89 (13.30-24.06)		8.70 (6.43–11.76)	

NOTE: IPD (invasive pneumococcal disease); PPsV23 (23-valent pneumococcal polysaccharide vaccine); PCV13 (13-valent pneumococcal conjugate vaccine); HR (hazard ratio); CI (confidence interval)

In both models (Tables 5 and 6) increasing age, sex male and history of previous IPD or all-cause pneumonia appeared as statistically significant predictors of suffering PP. Vaccinations’ history did not appear associated with reduced risk.

## Discussion

This large population-based cohort study investigated population-based incidence and risk of hospitalisation from PP among middle-aged and older adults with and without specific underlying medical conditions (including theoretically preventive or predisposing conditions), examining the effect of specific/individual and concurrent multiple risk conditions in the risk of suffering PP. Importantly, the study was conducted throughout 2017–2018, early period after universal free (publicly funded) PCV13 approval for all infants on June 2016 in Catalonia (a setting where PPsV23 for elderly people and PCV13 for high-risk adults are implemented since 1999 and 2012 respectively) [14].

As main findings, our data shows that the burden of hospitalised PP among adults over 50 years in Catalonia during 2017–2018 was moderate overall (with a global IR of 90.7 cases per 100,000 person-years), while considerably larger IRs appeared in very elderly individuals (i.e., 80 years or older), persons with history of hospitalisation from IPD or all-cause pneumonia in previous two-years, patients with immunocompromising conditions or chronic pulmonary/respiratory diseases and those with multi-comorbidity (i.e., two or more baseline risk conditions). In the multivariable analyses (apart of increasing age and history of prior IPD or all-cause pneumonia) major underlying conditions associated with increasing risk of PP were HIV-infection (which increased 6.8 times the adjusted risk of PP) followed by haematological neoplasia and chronic pulmonary/respiratory disease (both increasing approximately 2.7 times the adjusted risk of PP). Other conditions associated with a significant increased multivariable-adjusted risk of PP (with a range of 1.16–1.84 times increasing risk) were sex male, solid cancer, immunosuppressive therapy, renal disease, liver disease, heart disease, diabetes mellitus, alcoholism and smoking. Multi-comorbidity increased substantially the risk of PP, with multivariable-adjusted HRs increasing since 1.76 in persons with one comorbidity alone up to 3.10, 4.66, 7.07 and 8.70 for persons with 2, 3, 4 and 5 or more comorbidities, respectively.

Global incidence in this study is in the low limit of IRs reported for PP among adults in European settings (where IRs between 16 and 3581 hospitalised PP cases per 100,000 person-years have been published [6]. CFR in the present study (7.6%) may be considered intermediate since CFRs around 5–10% are commonly described for PP cases in older adults. [4–6] Nevertheless, if we compare our results with data observed in the same population during 2015–2016 (82.8 hospitalised PP cases per 100,000 person), [11, 21] we observe a little increase of PP burden despite increasing anti-pneumococcal vaccination coverage (especially in children) across this time period [22]. On this concern, public health impact of anti-pneumococcal vaccination programmes in adults remains a controversial issue at present. [12, 23–27] Indeed, new extended-valency conjugate vaccines have been developed to replace the “old” PPsV23/PCV13 vaccines [28].

In the present study, the vast majority (75.9%) of PP cases occurred among those cohort members with one or more underlying risk condition (who represented only a half [51.2%] of the total study cohort). This finding fits with other data reported in North America and Europe where the percentage of hospitalised adult IPD/PP cases with underlying medical conditions reached approximately 60% among people 18–64 years-old and 80% among persons 65 years or older. [2, 6, 29].

Regarding multi-comorbidity, our data fits with data reported in a literature review where prevalence of multi-comorbidity in pneumonia patients aged 65 years or older ranged from 23 to 98% for two or more comorbidities and from 18 to 89% for three or more comorbidities [10]. Our data underlines the capital role of multiple concurrent underlying risk conditions on the risk of suffering pneumonia. [6, 10].

As expected, immunocompromising conditions were related with a high excess risk of PP in the present study. Indeed, immunocompromised subjects, who were approximately 10% (9.9%) of the total study cohort, accounted for 24.4% of the total PP cases, supporting that immunocompromising conditions are major risk factors for pneumococcal disease (i.e., for both IPD/PP, not only for IPD). [2, 6, 7].

Cohort member classified as baseline at-risk subjects (38.9% of the total cohort) accounted for 51.5% of all PP cases in the study. Within at-risk subjects, the excess risk was highest for those with chronic pulmonary/respiratory disease, who were less than a 10% (9.7%) of the total study cohort but they suffered almost a third (32.5%) of the total PP cases and suffered an incidence/risk near to immunocompromised subjects. This result is in accordance with data reported in other studies and underlines the important role of chronic pulmonary/respiratory disease as predisposing factor to suffer pneumococcal infection. [2, 6, 7, 10]

As major strengths in this study we note that it’s a population-based design and the large size of the study cohort (more than 2 million people ≥ 50 years-old which represented almost 75% of the overall Catalonian inhabitants in this age strata) [13].We also note the use of survival analysis methods to estimate accurately incidence and risk of PP adjusted for major underlying medical conditions (including single high-risk or at-risk conditions and coexisting multi-comorbidities. Prevalence of chronic illnesses among patients with IPD/PP have been reported in numerous hospital case-series studies, but population-based data on this concern is limited [6].

Regarding smoking, a possible underestimation risk for PP is likely considering that there are few smoking people over 64 years old but exists a lot ex-smoker in this age strata (and also some smokers have died before this age). Regarding pneumococcal and influenza vaccinations, it must also be noted that people with comorbidities have a higher probability of vaccination and this may underestimate the possible preventive role for both vaccines.

As limitation, we assumed that hospital discharge ICD diagnose coding was correct and assuming that validation of the diagnosis was not feasible because of the study design and sample size. Regarding pneumococcal and influenza vaccinations, it must also be noted that people with comorbidities have a higher probability of vaccination and this may underestimate the possible preventive role for both vaccines.

We note that definition criteria for PP may vary between different studies, but we also note that using ICD codes to define PP, despite recognised limitations, [30] has been commonly used in many epidemiological studies evaluating this concern [6]. Despite other limitations, mainly linked to retrospective design and absence of specific microbiological data, we highlights that our study provides uncommon population-based data on incidence and risk of PP among healthy, at-risk and high-risk adults in the present era of multiple-valent pneumococcal conjugate vaccines. Although, these data are scarce in the literature, they are greatly needed to interpret possible direct and indirect impact of currently implemented childhood and adult pneumococcal vaccination programs. [9, 22] We underline that this study includes all diagnosed cases of PP, both confirmed (invasive/bacteremic) as well as presumptive cases (i.e., positive sputum culture or urinary antigen test with negative or not performed blood culture).

This is an important concern since bacteremic PP may represent only a little fraction (approximately 1/5) of the overall PP disease burden in adults. [3, 5, 6, 9] Nonbacteremic PP represents the vast majority of pneumococcal disease in adults and, therefore, it must be included in the analyses if the overall spectrum of the disease needs to be assessed.

## Conclusion

During 2017–2018 the overall burden of pneumococcal pneumonia requiring hospitalisation among middle-aged and older adults in Catalonia remained considerable, with an incidence of 90.7 hospitalised PP cases per 100,000 person-years and a global CFR of 7.6%. Besides increasing age and immunocompromising conditions, history of prior IPD/pneumonia, oldest age (i.e., 80 years or more), presence of chronic pulmonary disease and/or co-existing multi-comorbidity (i.e., two or more underlying risk conditions) are major independent risk factors for PP in adults (with an excess risk near to immunocompromised subjects). Redefining risk categories for PP, including all above-mentioned conditions into the high-risk category, could be necessary to improve prevention strategies in middle-aged and older adults.

## Appendix. Criteria used to define comorbidities/underlying risk conditions in the study population

The following comorbidities and underlying risk conditions were stablished according to the presence of ICD-10 codes [International Classification of Diseases, 10th Revision] registered in the electronic primary care medical records of each cohort member at baseline:

- **Chronic pulmonary/respiratory disease**: it included chronic bronchitis/emphysema (J41-J44), asthma (J45-J46) and/or other chronic pulmonary diseases (P27, E84, J47).
- **Chronic heart disease**: it included congestive heart failure (I50), coronary artery disease (I20-I22, I25) and/or other chronic heart diseases (I05-I08, I11,I35-I37,I42, I51.7).
- **Diabetes mellitus** (E10-E14).
- **Chronic liver disease**: it included chronic viral hepatitis (B18), cirrhosis (K74) and/or alcoholic hepatitis (K70)).
- **Alcoholism** (F10, G31.2, G62.1, G72.1, I42.6, K29.2, K70).
- **Smoking** (F17).
- **Anatomic or functional asplenia** (D57, D73, Q89).
- **Primary immunodeficiency** (D80-D84).
- **HIV infection** (B20-B24).
- **Chronic renal disease**: it included nephrotic syndrome (N04, N39.1) and severe chronic renal failure (N18-N19 with glomerular filtration rate ≤ 30 ml/min).
- **Cancer**: it included solid organ or haematological neoplasia (C00 to C97) diagnosed within previous 5 years.
- **Immunosuppressive therapy**: it included long-term immunosuppressive medication and/or radiotherapy in the previous 12 months (coded according to specific SIDIAP codes).

## Data Availability

These data have been obtained from the Catalonian Health Institute Information System for the Development of Research in Primary Care (SIDIAP). Interested authors might obtain SIDIAP data (previous ethics and scientific approval by the ethics and clinical research committee of the Primary Care Research Institute Jordi Gol/SIDIAP Jordi Gol) addressing purposes to the Institution. In accordance with current European and national law, the data used in this study is only available for the researchers participating in this study. Thus, we are not allowed to distribute or make publicly available the data to other parties. However, researchers from public institutions can request data from SIDIAP if they comply with certain requirements. Further information is available online (https://www.sidiap.org/index.php/menu-solicitudesen/application-proccedure) or by contacting SIDIAP (sidiap@idiapjgol.org), Clara Rodriguez-Casado (crodriguez@idiapjgol.info)).

## Abbreviations

CFR: Case-Fatality Rate
CI: Confidence Interval
CMBD: *Conjunto mínimo de base de datos*, Spanish hospital discharge codes
HIV: Human Immunodeficience Virus
HR: Hazard Ratio
ICD-10: International Classification of Diseases-10
ICS: Catalonian Health Institute, *Institut Català de la Salut*
IPD: Invasive pneumococcal disease
IR: Incidence rate
PCCs: Primary Care Centres (PCCs)
PCV: Pneumococcal Conjugate Vaccine
PP: Pneumococcal Pneumonia
PPV: Polysaccharide Pneumococcal Vaccine
SIDIAP: Catalonian Information System for Development of Research in Primary Care
